# Comparative Study of Regulatory T Cell Function of Human CD25^+^CD4^+^ T Cells from Thymocytes, Cord Blood, and Adult Peripheral Blood

**DOI:** 10.1155/2008/305859

**Published:** 2008-09-22

**Authors:** Wakae Fujimaki, Nozomu Takahashi, Kei Ohnuma, Masayoshi Nagatsu, Hiromi Kurosawa, Satoko Yoshida, Nam H. Dang, Takehiko Uchiyama, Chikao Morimoto

**Affiliations:** ^1^Division of Clinical Immunology, Advanced Clinical Research Center, Institute of Medical Science, University of Tokyo, Tokyo 108-8639, Japan; ^2^Department of Microbiology and Immunology, Tokyo Women's Medical University, Tokyo 162-8666, Japan; ^3^Human Medical Science Division, Kagawa Nutrition University, Saitama 350-0288, Japan; ^4^Department of Cardiovascular Surgery, Tokyo Women's Medical University, Tokyo 162-8666, Japan; ^5^Department of Obstetrics and Gynecology, Yoshida Clinic, Saitama 358-0054, Japan; ^6^Department of Hematologic Malignancies, Nevada Cancer Institute, Las Vegas, NV 89135, USA

## Abstract

CD25^+^CD4^+^ regulatory T cells suppress T cell activation and regulate multiple immune reactions in in vitro and in vivo studies. To define the regulatory function of human CD25^+^CD4^+^ T cells at various stages of maturity, we investigated in detail the functional differences of CD25^+^CD4^+^ T cells from thymocytes, cord blood (CB), and adult peripheral blood (APB). CB CD25^+^CD4^+^ T cells displayed low-FOXP3 protein expression level and had no suppressive activity. In contrast, CD25^+^CD4^+^ T cells from thymocytes or APB expressed high expression level of FOXP3 protein associated with significant suppressive activity. Although CB CD25^+^CD4^+^ T cells exhibited no suppressive activity, striking suppressive activity was observed following expansion in culture associated with increased FOXP3 expression and a shift from the CD45RA^+^ to the CD45RA^−^ phenotype. These functional differences in CD25^+^CD4^+^ T cells from Thy, CB, and APB hence suggest a pathway of maturation for Treg in the peripheral immune system.

## 1. INTRODUCTION

Naturally occurring regulatory T cells (Treg) are characteristically CD25^+^CD4^+^ and display strong suppressive activity against responder cell reactivity, although Treg themselves are in an anergic state, that is, they do not proliferate nor produce interleukin (IL)-2 on stimulation [1]. These Treg have pivotal role in controlling autoimmunity, tumor immunity, transplantation tolerance, maternal tolerance to the fetus, allergy, and microbial immunity [2–4]. CD25 is the first recognized marker for Treg, with CD152 (cytotoxic T lymphocyte-associated antigen-4) or glucocorticoid-induced tumor necrosis factor receptor-related protein (GITR) being also used [5–7]. However, since these molecules are expressed also on activated T cells, they are not specific markers for Treg. On the other hand, forkhead box P3 (FOXP3), a member of the well-known and diverse forkhead transcription factor family, is a master switch in Treg differentiation and function, while appearing to be a specific marker for Treg [8–12].

Several studies have shown that Treg are generated within the thymic medulla in response to high-affinity interactions with thymic epithelial cells [13], prior to exiting to the periphery. Recent work suggested that the Hassal's corpuscles have a critical role in the generation of human Treg within the thymus [14]. In contrast to knowledge regarding Treg development in thymus, the pathway of maturation for Treg in the peripheral immune system is still obscure. For this purpose, it is important to characterize the regulatory function of human CD25^+^CD4^+^ T cells at various stages of maturity. While the functions of Treg from thymocytes (Thy), cord blood (CB), or adult peripheral blood (APB) have been examined previously [15–19], it is difficult to concisely summarize the functional differences of Treg from these studies since the observed results were not consistent and since the functional assays for Thy, CB, and APB Treg were not simultaneously performed to facilitate comparison among these different components. Interestingly, a subset of naïve type Treg was identified recently in APB [17, 20],where it had been previously thought that the Treg population was of the CD45RO^+^ activated/memory subset. The naïve CD45RA^+^ Treg is predominant in the CB Treg population [18], and is prominent in young adults while decreasing with age [20]. However, details regarding the maturation process of naïve type APB Treg and CB Treg have not been heretofore conclusively clarified.

We previously examined the process involved in post-thymic maturation of conventional CD4^+^ T cells by investigating the antigen-reactivity status of CD4^+^ T cells from Thy, CB, and APB [21, 22]. Conventional CD4^+^ T cells migrating from the thymus appear to be required for the acquisition of full reactivity. The immaturity of conventional CD4^+^ T cells from CB is thought to affect the pathogenesis of infectious diseases [23, 24]. Since CB is used for stem cell transplantation, CB Treg function is especially important in understanding the low occurrence of graft-versus-host disease (GVHD) in this clinical setting [25, 26]. Furthermore, it is important to understand the unique immunological reactivity of the newborn. We have therefore focused our effort on determining the regulatory function of CD25^+^CD4^+^ T cells at various stages of maturity.

In the present study, we performed comprehensive comparative analysis of CD25^+^CD4^+^ T cells from APB, CB, and Thy through multicolored staining of cell surface and intracellular molecules, including FOXP3, and functional assays. Interestingly, the suppressive activity of fresh CB CD25^+^CD4^+^ T cells was lacking in contrast to that found in APB CD25^+^CD4^+^ T cells and Thy CD25^+^CD4^+^ T cells. However, expansion of the CB CD25^+^CD4^+^ T cells in vitro resulted in the restoration of superior suppressive activity to that of APB or Thy CD25^+^CD4^+^ T cells following expansion. Based on our data, we propose a maturation pathway for peripheral human CD25^+^CD4^+^ T cells and discuss the potential biological implications of the unique functions observed in the CB CD25^+^CD4^+^ T cells.

## 2. MATERIALS AND METHODS

### 2.1. Purification of CD25^+^ and CD25^−^CD4^+^ T cells

APB was obtained from healthy adult donors (24–52 years of age) and CB was obtained from healthy full-term neonates within 24 hours of delivery, after written informed consent was obtained. APB and CB mononuclear cells were isolated by Lymphoprep (Nycomed, Oslo, Norway) density gradient centrifugation. Single-cell suspensions of thymocytes were obtained from thymus fragments dissected from donors, ranging in age from 2 months to 10 years, during corrective cardiac surgery after obtaining written informed consent. CD25^+^CD4^+^ and CD25^−^CD4^+^ T cells were isolated by CD4^+^CD25^+^ regulatory T cell isolation kit (Miltenyi Biotec, Auburn, Calif., USA). The resulting purified CD25^+^CD4^+^ and CD25^−^CD4^+^ T cells contained <5% CD25^−^CD4^+^ T cells and 1% CD25^+^CD4^+^ T cells, respectively.

### 2.2. Isolation of CD45RA^+^ and RA^−^CD25^+^CD4^+^ T cells

To isolate CD45RA^+^CD25^+^CD4^+^, CD45RA^−^CD25^+^CD4^+^, and CD25^−^CD4^+^ T cells, CD4^+^ T cells were first purified by negative selection with the CD4 T cell Isolation kit II (Miltenyi Biotec). Bead-selected CD4^+^ cells were stained with anti-CD4-allophycocyanin (APC), anti-CD25-phycoerythrin (PE), and anti-CD45RA-fluoroscein isothiocyanate (FITC) antibodies (BD Biosciences), and sorted by a FACSAria cell sorter (BD Bioscience, Franklin Lakes, NJ, USA). After gating on the CD4^+^ lymphocytes, cells were separated into 3 tubes on the basis of CD45RA and CD25 expression.

### 2.3. Preparation of antigen-presenting cells

Autologous mononuclear cells were used as antigen-presenting cells. Prior to use as antigen-presenting cells, cells were irradiated at 30 gray with a *γ*-ray irradiator and treated with mitomycin C at 50 *μ*g/ml for 30 minutes at 37°C.

### 2.4. Proliferation assay

Responding CD25^−^CD4^+^ T cells (2 × 10^4^ cell/well) were stimulated with anti-CD3/CD28-monoclonal antibody (mAb-) coated beads (Dynal Biotech ASA, Oslo, Norway) in the presence of antigen-presenting cells (2 × 10^4^ cell/well) in 0.2-ml volumes in 96-well U-bottom plates for 96 hours. CD25^+^CD4^+^ T cells were added in graded numbers as indicated. Anti-CD3/CD28-mAb-coated beads as stimulants were added at a 1 : 10 bead-to-responder cell ratio. Culture media was RPMI 1640 supplemented with 10% fetal calf serum (FCS; Invitrogen, Carlsbad, Calif., USA). Cultures were pulsed with 37 kBq of ^3^H-thymidine for the last 16 hours of culture period. Results were expressed as the mean counts per minute of triplicate wells.

### 2.5. Cytokine measurement

Responding CD25^−^CD4^+^ T cells (2 × 10^5^ cells/well) were stimulated with anti-CD3/CD28-mAb-coated beads in the presence of antigen-presenting cells (2 × 10^5^ cells/well) in 0.2-ml volumes in 96-well U-bottom culture plates. Either 2 × 10^5^ CD25^+^CD4^+^ T cells or none were then added. Anti-CD3/CD28-mAb-coated beads as stimulants were added at a 1 : 3 bead-to-responder cell ratio. After 48 hours of culture, the supernatants were collected from each well, and the levels of IL-2 or those of transforming growth factor (TGF)-*β* in the culture supernatants were measured by enzyme-linked immunosorbent assay (ELISA; BD Biosciences). For the profiling of cytokine production, IL-2, IL-4, IL-5, IL-10, interferon (IFN)-*γ*, and tumor necrosis factor (TNF) *α* levels in supernatants were measured using a cytometric bead array kit (BD Bioscience), according to the manufacturer instructions. All data were presented as picograms per mL.

### 2.6. Flow cytometric analysis

For phenotypic analysis of mononuclear cells and thymocytes, cells were stained with several combinations of mAbs as shown in Table 1: anti-CD45RO-FITC (UCHL1), anti-CD45RA-FITC (HI100), anti-CD62L-FITC (Dreg56), anti-CD152-PE (BNI3), anti-CD8-peridinin chlorophyll protein-cycrome 5.5 (PerCP-Cy5.5) (SK1), anti-CD3-PE-Cy7 (SK7), anti-CD4-PE-Cy7 (SK3), anti-CD62L-APC (Dreg56), anti-CD25-APC (M-A251), anti-CD4-APC-Cy7 (RPA-T4), anti-CD8-pacific blue (RPA-T8), and anti-CD152-biotin (BNI3.1, BD Biosciences), anti-CD25-PE (4E3, Miltenyi Biotec, Auburn, Calif., USA), anti-FOXP3-PE (PCH101, eBioscience, San Diego, CA), and anti-CD45RA-pacific blue (MEM-56, Caltag, Burlingame, Calif., USA). Biotin conjugates were developed with streptoavidin-PE-Cy7 (BD Biosciences). For cell surface staining, all Abs were added to cells except propidium iodide (PI), and cells were stained for 20 minutes. PI was added to unfixed samples just prior to FACS analysis to exclude dead cells. When performing intracellular staining for FOXP3 and/or CD152, cell-surface staining was first completed, and cells were fixed and permeabilized for intracellular staining. For upper intracellular staining pattern in Table 1, 1% formaldehyde and 0.5% saponin were used for fixation and permeabilizaion, respectively. For lower intracellular staining pattern in Table 1, FOXP3 staining set (eBioscience) was used according to the manufacturer instructions. Ethidium monoazide bromide (EMA) was added prior to fixation to exclude dead cells. Stained cells were examined by eight-color flow cytometric analysis using a FACSAria cell sorter (BD Bioscience). Data analysis was performed using FlowJo software (Treestar, San Carlos, Calif., USA).

For phenotypic confirmation of the CD25^+^CD4^+^ and CD25^−^CD4^+^ T cell preparation, cells were stained for three-color analysis with several combinations of the following mAbs: anti-CD4-FITC (RPA-T4), anti-CD8-PE (RPA-T8), anti-CD45RA-FITC (HI100) and anti-CD45RO-PE (UCHL1, BD Biosciences), anti-CD25-PE (4E3, Miltenyi Biotec), and anti-CD3-phycoerythrin cyanin 5 (PC5) (UCHT1, Immnunotech, Marseille, France). Analysis was performed on a FACSCalibur using Cellquest software.

### 2.7. Western blotting

Cells (2 × 10^5^/sample) were lysed in cell lysis buffer (containing 10 mM Tris-HCl (pH7.4), 1% Nonidet P-40, 0.1% sodium deoxycholate, 0.1% sodium dodecyl sulfate (SDS), 0.15 M NaCl, 1 mM ethylenediaminetetraacetic acid (EDTA), 10 *μ*g/ml aprotinin, and 300 *μ*g/ml phenylmethylsulfonyl fluoride). The cell lysates were subjected to a 10% SDS-polyacrylamide gel electrophoresis under reducing conditions, transferred to a polyvinylidene difluoride membrane, and immunoblotted with mouse anti-FOXP3 Ab (Abcam, Cambridge, Mass., USA) followed by horseradish peroxidase-conjugated antimouse IgG (GE Healthcare UK Ltd. Buckinghamshire, UK) as the second Ab. The immunoblots were developed with Western Lightening Chemiluminescence Reagent Plus (PerkinElmer Life Sciences. Inc., Boston, Mass., USA). As positive control for Western blot analysis, recombinant human FOXP3 fusion protein was produced by cloning full length human FOXP3, which was generated by PCR, into the pcDNA4/HisMax vector (Invitrogen).

### 2.8. Expansion culture of CD25^+^CD4^+^ T cells

Isolated CD25^+^CD4^+^ T cells were cultured at 1 × 10^6^ cells per milliliter with anti-CD3/CD28 mAb-coated beads at a 1 : 10 bead-to-cell ratio and recombinant human IL-2 (Shionogi, Osaka, Japan) at 100 U/ml. For these cultures, since the addition of irradiated antigen-presenting cells was not helpful for expansion, as reported previously [27], antigen-presenting cells were not utilized. Cell cultures were split as needed. For the last 24 hours of the culture period, cells were cultured without IL-2. Culture media was RPMI 1640 supplemented with 10% FCS, penicillin, and streptomycin. Recovered cells were subjected to Percoll (density = 1.050) centrifugation. Viable cells were obtained under density of 1.050. 

## 3. RESULTS

### 3.1. Phenotypic comparison of CD25^+^CD4^+^ T cells populations from APB, CB, and Thy

**Figure 1:**
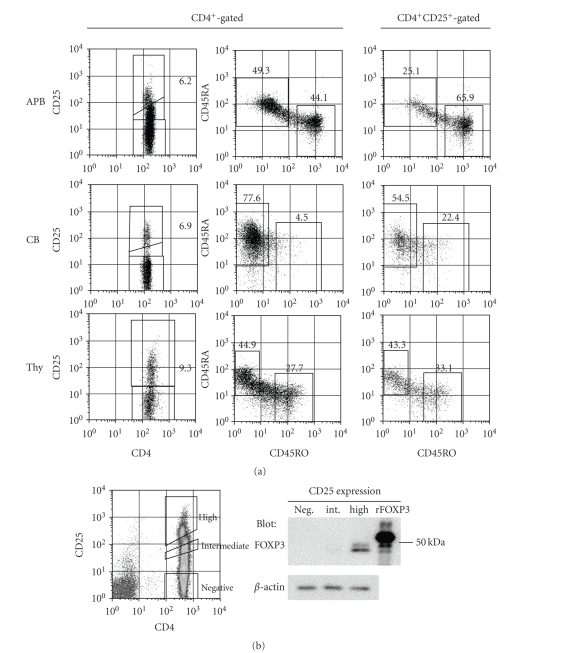
*Predominance of CD45RA^+^ type CD25^+^CD4^+^ T cells in CB, and existence of CD45RA^+^ and CD45RO^+^ type CD25^+^CD4^+^ T cells in APB and Thy.* (a) Mononuclear cells or thymocytes were multistained with anti-CD3, anti-CD4, anti-CD8, anti-CD25, anti-CD45RA, anti-CD45RO, anti-CD62L mAbs, and PI (Table 1). The profiles were gated for PI^−^CD3^+^CD8^−^CD4^+^ cells (left and middle columns) or CD3^+^CD8^−^CD4^+^CD25^+^ cells (right column), and expression of CD45RA versus CD45RO was illustrated. Data are representative of 3 different donors. (b) APB CD4^+^ T cells were stained with anti-CD4 and anti-CD25 mAbs. On the basis of CD4 and CD25 expression, CD25^high^ CD4^+^ cells, CD25^intermediate^ CD4^+^ cells, and CD25^negative^ CD4^+^ cells were sorted. Each cell population was lysed and immunoblotted with anti-FOXP3 or anti-*β*-actin Ab. FOXP3 in human PBMC was detected at a lower position than the recombinant human FOXP3 fusion protein used as positive control, since the recombinant human FOXP3 fusion protein contains approximately 4 kDa of additional peptides such as the 6xHis tag, Xpress epitope tag, and enterokinase recognition site, as described in Section 2 .

To compare the phenotype of CD25^+^CD4^+^ T cells from APB, CB, and Thy, we first performed eight-color staining analysis to evaluate the T cell phenotype of each compartment (Figure 1(a)). Left and middle columns were gated for PI^−^CD3^+^CD8^−^CD4^+^ cells and right column was gated additionally for cells shown in the left column that highly expressed CD25. As shown in the upper row of the left column of Figure 1(a), APB CD4^+^ T cells were divided into three populations differing in CD25 expression levels, which were high, intermediate, and negative. Since FOXP3 protein level is a reliable marker for defining Treg, we next examined its expression in these T cell populations. As shown in Figure 1(b), Western blotting for FOXP3 protein expression confirmed that CD25^high^ CD4^+^ T cells expressed the highest level of FOXP3 protein, with this population being Treg. In CB and Thy, since cells expressing intermediate level of CD25 were not so prominent, it was relatively easy to identify the Treg population as being cells expressing high levels of CD25^+^ (Figure 1(a), middle and bottom rows of left column). The percentages of CD4^+^ T cells with high CD25 expression were 5 ± 1% in APB (mean ± SD of 10 different donors), 7 ± 2% in CB (*n* = 10), and 9 ± 4% in Thy (*n* = 4). Although CD45RA^+^ and CD45RO^+^ were abundantly expressed on APB CD4^+^ T cells (Figure 1(a), middle of upper row), CD45RO^+^ was predominantly found on the gated CD25^high^ CD4^+^ T cell subset (Figure 1(a), right of upper row; 27 ± 11% CD45RA^+^ and 66 ± 6% CD45RO^+^, *n* = 3). In CB, the CD45RA isotype was predominant in not only CD4^+^ T cells (Figure 1(a), middle of second row) but also CD25^+^CD4^+^ T cells (Figure 1(a), right of second row; 62 ± 11% CD45RA^+^ and 21 ± 2% CD45RO^+^, *n* = 3). In thymocytes, CD25^+^CD4^+^ T cells expressed both CD45RA and CD45RO isotypes (Figure 1(a), right of bottom row; 35 ± 10% CD45RA^+^ and 46 ± 13% CD45RO^+^, *n* = 3), similar to thymic total CD4^+^ T cells (Figure 1(a), middle of bottom row). Meanwhile, all of the CD25^+^CD4^+^ T cells expressed CD62L, regardless of their expression of CD45RA^+^ or CD45RO^+^ (data not shown). It was clear from our present data that CD45RA^+^ type CD25^+^CD4^+^ T cells was predominant in CB while both CD45RA^+^ and CD45RO^+^ type CD25^+^CD4^+^ T cells existed in APB and Thy.

### 3.2. Lack of suppressive function of CB CD25^+^CD4^+^ T cells

**Figure 2:**
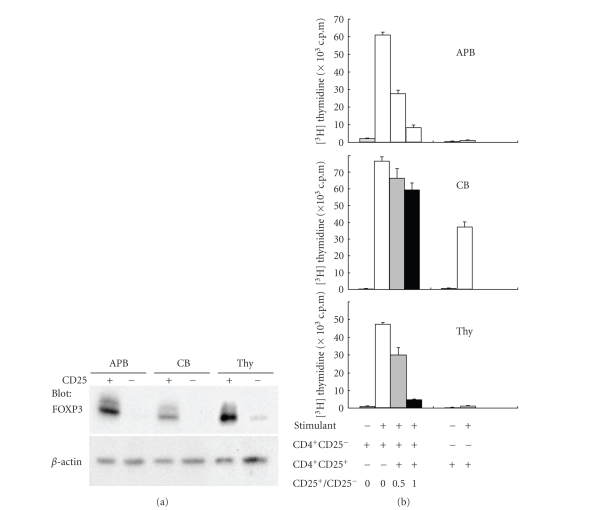
*Lack of suppressive activity and absence of an anergic state by CB CD25^+^CD4^+^ T cells.* CD4^+^CD25^+^ and CD4^+^CD25^−^ T cells were purified by a magnetic microbead system. (a) Cells were lysed and immunoblotted with anti-FOXP3 Ab (top panel) or anti-*β*-actin Ab (bottom panel). (b) Cells were stimulated with anti-CD3/CD28-mAb-coated beads under the indicated conditions. Cell proliferation was assessed by ^3^H-thymidine uptake on day 4. Data are representative of 5 different donors.

**Figure 3:**
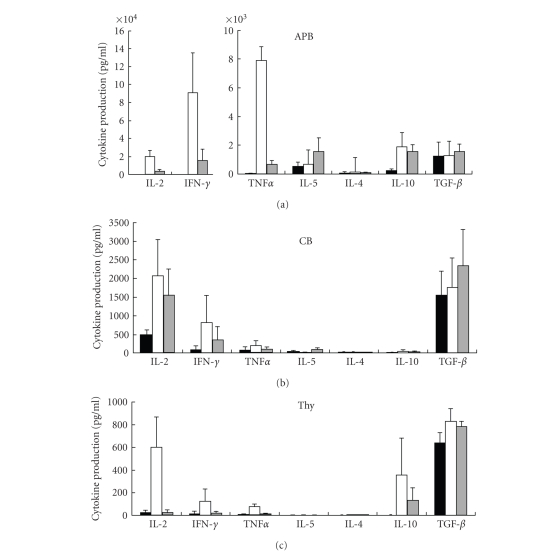
*Suppressive effect of CD4^+^CD25^+^ T cells on cytokine production by CD4^+^CD25^−^ responder cells.* CD4^+^CD25^+^ T cells and CD4^+^CD25^−^ responder cells were prepared by a magnetic microbead system. Cells were stimulated with anti-CD3/CD28-mAb-coated beads in the presence of antigen-presenting cells. The supernatants were removed after 48 hours of incubation with either CD4^+^CD25^+^ T cells (black bars), CD4^+^CD25^−^ responder cells (white bars), and cocultured cells (gray bars). TGF-*β* level in supernatants was measured using ELISA, and levels of other cytokines were measured using a cytometric bead array kit. Data are mean ± SD of 3 independent experiments.

We next determined the in vitro regulatory function of the CD25^+^CD4^+^ populations derived from APB, CB, and Thy. CD25^+^CD4^+^ T cells and CD25^−^CD4^+^ T cells were separated using a magnetic microbead system, with Western blotting for FOXP3 confirming that Treg existed predominantly in the CD25^+^CD4^+^ T cell preparations from APB, CB, and Thy (Figure 2(a)). It was noted that FOXP3 protein levels were different among CD25^+^CD4^+^ T cells from APB, CB, or Thy with the expression level being very weak in CD25^+^CD4^+^ T cells from CB. We next evaluated the suppressive activity of CD25^+^CD4^+^ T cells on autologous CD25^−^CD4^+^ T cell proliferation induced by anti-CD3/CD28 mAb-coated beads, as well as the proliferative activity of CD25^+^CD4^+^ T cells themselves by thymidine uptake (Figure 2(b)). CD25^+^CD4^+^ T cells from APB or Thy suppressed proliferation of responding CD25^−^CD4^+^ T cells in the presence of autologous antigen-presenting cells in a dose-dependent fashion, whereas a lack of suppression was observed in CB CD25^+^CD4^+^ T cells. Additionally, CB CD25^+^CD4^+^ T cells, but not APB or Thy CD25^+^CD4^+^ T cells, proliferated well upon stimulation with anti-CD3/CD28 mAb-coated beads. We simultaneously evaluated IL-2 production status of CD25^+^CD4^+^ T cells and responding CD25^−^CD4^+^ T cells from APB, CB, and Thy (Figure 3). APB and Thy CD25^+^CD4^+^ T cells produced much lower levels of IL-2 than responding APB and Thy CD25^−^CD4^+^ T cells. On the contrary, CB CD25^+^CD4^+^ T cells did produce substantial level of IL-2. Absolute level of IL-2 production from responding CD25^−^CD4^+^ T cells was highest in those from APB, followed by those from CB, being lowest in those from Thy. In summary, our data indicated that CD25^+^CD4^+^ T cells from CB were not in an anergic state and did not possess biologically significant suppressive activity, in marked contrast to APB or Thy CD25^+^CD4^+^ T cells. In addition to IL-2 production, we examined the production of various cytokines by CD25^−^CD4^+^ T cells and CD25^+^CD4^+^ T cells as well as the suppressive effect of CD25^+^CD4^+^ T cells on cytokine productions by responding CD25^−^CD4^+^ T cells (Figure 3). It should be noted that responding CD25^−^CD4^+^ T cells from CB and Thy did not produce high levels of cytokines except the suppressive cytokine TGF-*β*, as compared to those produced by APB. In particular, CB and thymic cells did not produce detectable levels of IL-4 or IL-5. While CD25^+^CD4^+^ T cells from APB and Thy produced much lower levels of IL-2, IFN-*γ* or TNF*α* as compared to those produced by responding cells, CB CD25^+^CD4^+^ T cells secreted substantial levels of these cytokines. Furthermore, CD25^+^CD4^+^ T cells from APB, CB, or Thy did not produce high level of the suppressive cytokine IL-10, but secreted high level of TGF-*β*. While CD25^+^CD4^+^ T cells suppressed the production of IL-2, IFN-*γ*, and TNF*α*, this suppressive activity was much weaker in CB compared to APB and Thy. Notably, CD25^+^CD4^+^ T cells from all three compartments did not suppress the production of TGF-*β*.

### 3.3. Weak expression FOXP3 and CD152 on CD25^+^CD4^+^ T cells from CB

**Figure 4:**
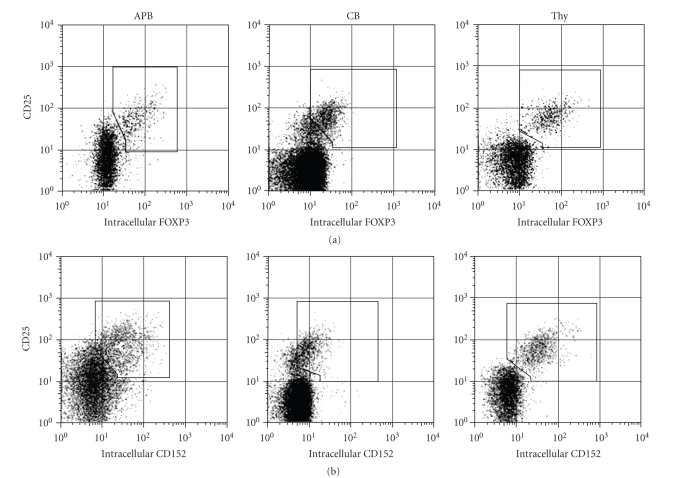
*Weak expression of FOXP3 and CD152 proteins in CB CD25^+^CD4^+^ T cells.* Mononuclear cells or thymocytes were multistained with anti-CD4, anti-CD8, anti-CD25, anti-CD45RA, antI-FOXP3, and anti-CD152 Abs (Table 1). The profiles were gated for CD8^−^CD4^+^, and showed expression of CD25 versus endogenous FOXP3 (top panel) or CD152 (bottom panel). Additional gating was set on FOXP3 or CD152 positive cells. Data are representative of 3 different donors.

The expression of FOXP3 and CD152 is associated closely with Treg function [6, 8–10, 12, 28, 29]. As shown in Figure 2(a), FOXP3 protein was detected only in CD25^+^CD4^+^ T cells from all three compartments, although being weaker in CB CD25^+^CD4^+^ T cells than APB or Thy CD25^+^CD4^+^ T cells. We next analyzed endogenous FOXP3 and CD152 protein expression by flow cytometry (Figure 4). Mononuclear cells and thymocytes were evaluated by multicolor analysis. FOXP3 expression was analyzed with data from lower intracellular staining pattern in Table 1, and CD152 expression with data from upper intracellular staining pattern. We found that protein expression level of FOXP3 and CD152 correlated with that of CD25, with higher CD25 expression associated with higher endogenous level of FOXP3 or CD152, and demonstrated that the mean fluorescence level of FOXP3 (99 ± 33 APB, 25 ± 10 CB and 82 ± 42 Thy, *n* = 3) or CD152 (46 ± 31 APB, 16 ± 1 CB and 55 ± 7 Thy, *n* = 3) was weaker in CB than that in APB and Thy. In summary, the protein expression level of both FOXP3 and CD152 was clearly weaker in CD25^+^CD4^+^ T cells from CB than those from APB and Thy, consistent with the lack of suppressive activity of CB CD25^+^CD4^+^ T cells.

### 3.4. CD45 isotype and Treg function

**Figure 5:**
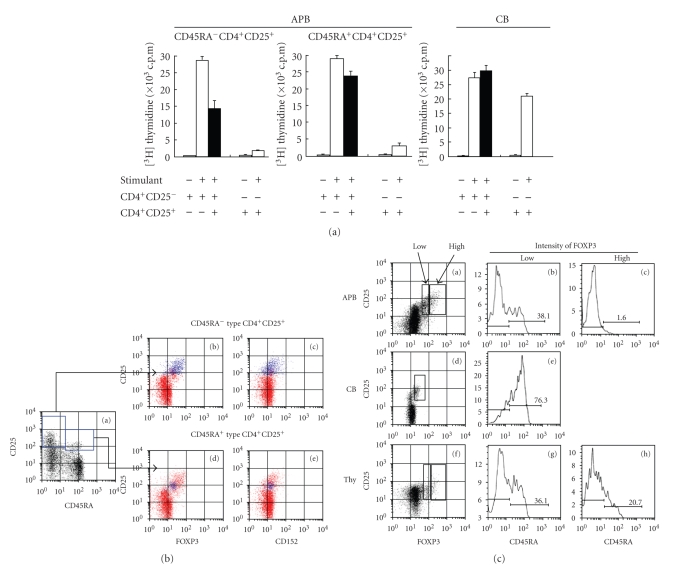
*Weak suppressive activity and expression of FOXP3 and CD152 proteins by CD45RA^+^ type APB CD25^+^CD4^+^ T cells.* (a) CD45RA^+^CD25^+^CD4^+^, CD45RA^−^CD25^+^CD4^+^, and CD25^−^CD4^+^ cells were sorted and incubated under the indicated conditions as described in Section 2. Cell proliferation was assessed by ^3^H-thymidine uptake on day 4. Data are representative of 3 different donors. (b) Mononuclear cells from APB were multistained with anti-CD4, anti-CD8, anti-CD25, anti-CD45RA, anti-FOXP3, and anti-CD152 Abs as described in Table 1. The profiles were gated initially for CD8^−^CD4^+^, then CD45RA^−^ type or CD45RA^+^ type CD25^+^CD4^+^ T cells were gated based on the expression of CD45RA and CD25 in panel (a). The expressions of CD25 versus endogenous FOXP3 or CD152 in CD45RA^−^ type (panels (b) and (c)) or CD45RA^+^ type CD25^+^CD4^+^ T cells (panels (d) and (e)) were merged as blue dots to those in CD8^−^CD4^+^ cells (red dots in panels (b)–(e)). Data are representative of 10 different donors. (c) Mononuclear cells or thymocytes were multistained as described in Figure 4(b). On the basis of FOXP3 expression, FOXP3^high^ or FOXP3^low^ was gated (panels (a), (d), (f)). In each population, histogram of CD45RA expression was shown (panels (b), (c), (e), (g), (h)).

Our data showed that CD25^+^CD4^+^ T cells from CB, which preferentially expressed the CD45RA^+^ phenotype, showed a lack of suppressive activity compared to APB CD25^+^CD4^+^ T cells, which contained both CD45RA^+^ and CD45RO^+^subsets. To define whether CD45RA^+^-expressing APB CD25^+^CD4^+^ T cells and CB CD25^+^CD4^+^ T cells exhibited the same levels of suppressive activity, we compared the suppressive activity of CD45RA^+^ type APB CD25^+^CD4^+^ T cells, CD45RA^−^ type APB CD25^+^CD4^+^ T cells, and CB CD25^+^CD4^+^ T cells (Figure 5(a)). CD45RA^−^ type APB CD25^+^CD4^+^ T cells, which mainly consisted of CD45RO^+^CD25^+^CD4^+^ T cells, showed high suppressive activity and were themselves in an anergic state. Meanwhile, the CD45RA^+^ type APB CD25^+^CD4^+^ T cells showed less suppressive activity than the CD45RA^−^ type APB CD25^+^CD4^+^ T cells and were found mostly in an anergic state, although capable of proliferation to some extent. However, CB CD25^+^CD4^+^ T cells did not show any suppressive activity and were not in an anergic state.

Since it has been reported that suppressive activity of Treg is associated with its expression of FOXP3 protein [8, 9], we next assessed the endogenous expression of FOXP3 and CD152 in CD45RA^+^ and CD45RA^−^ type CD25^+^CD4^+^ T cells from APB using the lower intracellular staining pattern in Table 1. Figure 5(b) shows the expression profiles of CD45RA versus CD25 (square gates in panel (a)), FOXP3 versus CD25 (blue dots in panels (b) and (d)), or CD152 versus CD25 (blue dots in panels (c) and (e)) among CD8^−^CD4^+^ T cells (red dots in panels (b)–(e)). APB CD45RA^−^ type CD25^+^CD4^+^ T cells showed a wide range of FOXP3 (MFI = 86; blue dots in panel (b) of Figure 5(b)) and CD152 (MFI = 40; blue dots in panel (c) of Figure 5(b)) expression, while CD45RA^+^ type CD25^+^CD4^+^ T cells exhibited only weak expression of FOXP3 (MFI = 33; blue dots in panel (d) of Figure 5(b)) and CD152 (MFI = 19; blue dots in panel (e) of Figure 5(b).

To further assess the relationship between CD45 isotype expression and Treg, we analyzed CD45RA expression in relation to FOXP3 expression (Figure 5(c)). APB CD25^+^CD4^+^ T cells expressing high level of FOXP3 (panel (a)) were CD45RA^−^ (panel (c)), while CD25^+^CD4^+^ T cells with low FOXP3 expression (panel (a)) consisted of both CD45RA^+^cells and CD45RA^−^ cells (panel (b)). Meanwhile, CB CD25^+^CD4^+^ T cells were weakly FOXP3 positive (panel (d)) and were mainly CD45RA^+^cells (panel (e)). Thymic CD25^+^CD4^+^ T cells contained both CD45RA^+^ and CD45RA^−^ cells, regardless of the intensity of FOXP3 expression (panels (f)–(h)).

Taken together, our data suggested that Treg function was correlated with the FOXP3 expression level and CD45 isotype.

### 3.5. Restoration of suppressive activity in expanded CB CD25^+^CD4^+^ T cells

**Figure 6:**
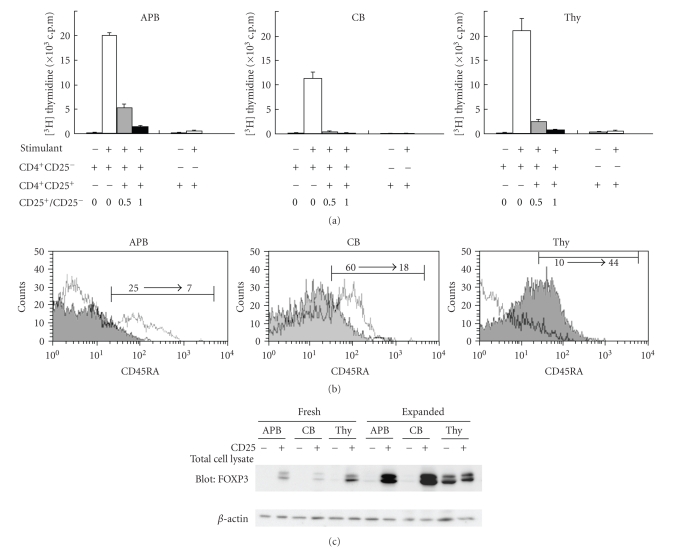
*Restoration of suppressive activity in expanded CB CD25^+^CD4^+^ T cells.* (a) Freshly isolated CD4^+^CD25^+^ cells were used as Treg in Figure 2(b), and expanded as described in Section 2. Freshly isolated CD25^−^CD4^+^ T cells were used as responder cells in both primary and secondary assays without expansion. Cell proliferation was assessed by ^3^H-thymidine uptake on day 4. Data are obtained by experiments with samples of 3 different donors. (b) Freshly isolated CD4^+^CD25^+^ cells or expanded CD4^+^CD25^+^ cells were stained with anti-CD3, CD25, and CD45RA, and analyzed using FACSCalibur. The cells were gated for CD3^+^CD25^+^ cells and the expression of CD45RA was compared between freshly isolated CD4^+^CD25^+^ cells (open histogram) and expanded CD4^+^CD25^+^ cells (closed histogram). Data are representative of 3 different donors. (c) Expression of FOXP3 protein was compared between freshly isolated cells and expanded cells by Western blot. Data are representative of 3 different donors.

Freshly isolated CB CD25^+^CD4^+^ T cells, exhibiting the CD45RA^+^ phenotype, lacked suppressive activity in our experimental system. We next examined the suppressive activity, CD45RA expression, and FOXP3 protein level of CD25^+^CD4^+^ T cells prior to and following in vitro activation. As shown in Figure 6(a), expanded CB CD25^+^CD4^+^ T cells showed excellent suppressive activity and were in a complete anergic state, while fresh CB CD25^+^CD4^+^ T cells were not in an anergic state and did not possess any suppressive activity (Figure 2(b)). Likewise, expanded APB and Thy CD25^+^CD4^+^ T cells displayed greater suppressive activity than freshly isolated CD25^+^CD4^+^ T cells, although the difference was not as prominent as compared to that observed in CB (Figure 6(a)). Notably, the phenotype of Thy CD25^+^CD4^+^ T cells shifted from CD45RA^−^ to CD45RA^+^ following expansion, whereas CD25^+^CD4^+^ T cells from CB and APB shifted from CD45RA^+^ to CD45RA^−^ (Figure 6(b)). Meanwhile, FOXP3 expression increased following the expansion of CD25^+^CD4^+^ T cells from APB, CB, and Thy (Figure 6(c)). The enhancement in FOXP3 level was especially prominent in the expanded CB CD25^+^CD4^+^ T cells population, consistent with the marked increase in suppressive activity (Figures 6(a) and 6(c)). Of note was also the fact that the responding CD25^−^CD4^+^ cells from Thy became FOXP3 positive following the expansion in culture (Figure 6(c)). Our findings thus indicated that expansion of CB CD25^+^CD4^+^ T cells resulted in the restoration of potent suppressive activity and an anergic state, associated with a concomitant increase in FOXP3 expression and a shift from the CD45RA^+^ to CD45RA^−^ phenotype.

## 4. DISCUSSION

To define the regulatory function of human Treg in each stage of maturation, we compared the phenotype and regulatory function of CD25^+^CD4^+^ T cells from Thy, CB, and APB. In this study, we have demonstrated that the suppressive function of fresh CB CD25^+^CD4^+^ T cells was lacking in contrast to APB CD25^+^CD4^+^ T cells and Thy CD25^+^CD4^+^ T cells, and subsequent expansion of the CB CD25^+^CD4^+^ T cells population resulted in the restoration of its suppressive activity. The observed difference in Treg function in CD25^+^CD4^+^ T cells from Thy, CB, and APB was associated with alteration in the CD45 isotype and FOXP3 expression level.

We demonstrated herein that the lack of functional activity was solely observed in CB CD25^+^CD4^+^ T cells, which weakly expressed FOXP3 and CD152, showed no suppressive activity and were not in an anergic state (Figures 2 and 4). While previous studies have examined the function of Thy, CB, or APB Treg, interpretation of the observed results may be complicated by differences in experimental conditions [15–17, 19]. For example, Wing et al. reported that CB Treg suppressed proliferation by CD25^−^ T cells stimulated with myelin oligodendrocyte glycoprotein (MOG) but not with staphylococcus enterotoxin B (SEB) [19], while an earlier study by the same group showed that CD25^+^ Treg did not suppress MOG-induced proliferation [30]. On the other hand, other groups demonstrated sufficient suppressive activity by CB Treg as compared to APB Treg following stimulation with PHA or anti-CD3 mAb [16, 17]. Moreover, Thornton et al. showed that fresh CB Treg did not display suppressive activity in mixed lymphocyte reaction system in contrast to the suppressive activity seen in CB Treg cultured with ovalbumin [15]. Therefore, it is unclear whether or not antigen-specific effector and regulatory T cells are present in CB, or whether SEB provides a T cell receptor (TCR-) mediated signal of sufficient strength to render CD25^−^ T cells refractory to inhibition by CD25^+^ Treg. To address these issues, our present work compared the functions of CD25^+^CD4^+^ T cells from the Thy, CB, and APB compartments together in the same experimental system, using the combination of anti-CD3-mAb plus anti-CD28-mAb as the standard TCR stimulants. However, we could not formally exclude the possibility that nonregulatory CD25^+^ T cells in isolated Treg fraction, or noneffector CD25^−^ T cells in isolated responding CD25^−^ T cell fraction, might contaminate the cell fraction of interest at an undetectable level, since isolation of cells was conducted by magnetic bead separation.

Cytokine profiles of APB Treg were previously reported by some investigators [31, 32], and other investigators reported production of IFN-*γ* and IL-10 by CB Treg or Thy Treg [19]. In general, previous studies demonstrated that Treg did not produce IL-2 and IFN-*γ*, as was also the case with our present data with APB and Thy CD25^+^CD4^+^ T cells (Figure 3). On the other hand, we observed that CB CD25^+^CD4^+^ T cells produced substantial levels of IL-2 and IFN-*γ* and did not inhibit cytokine production by responder cells sufficiently, consistent with our observation of their lack of suppressive activity on responder cell proliferation. Notably, the suppressive cytokine TGF-*β* was produced by both CD25^+^CD4^+^ T cells and responder cells in all of the APB, CB, and Thy compartments, while IL-10 and TGF-*β* production was prominent in Thy. Although it might be argued that various cytokines may be produced by T cells in response to different in vitro stimulants, our observation shown in Figure 3 suggests that the Treg population may be maintained in some circumstances by IL-10 and/or TGF-*β*, consistent with previous work showing a role for TGF-*β* in the in vivo expansion of Treg [33].

As shown in Figure 5(a), we demonstrated that the suppressive activity of CD45RA^+^ type APB CD25^+^CD4^+^ T cells was between that of CD45RA^−^ type APB CD25^+^CD4^+^ T cells and CB CD25^+^CD4^+^ T cells. Furthermore, cellular expression of the FOXP3 protein in CD45RA^+^ type APB CD25^+^CD4^+^ T cells was low and clearly different from that of CD45RA^−^ type APB CD25^+^CD4^+^ T cells (Figure 5(b)). These observations suggested that CD45RA^+^ type APB CD25^+^CD4^+^ T cells were different compared with CB CD25^+^CD4^+^ T cells, being mostly CD45RA^+^, as well as being different compared with CD45RA^−^APB CD25^+^CD4^+^ T cells. Wing et al. have shown that CD45RA^+^CD25^+^ T cells are comprised of approximately two-third of the CD25^+^ T cells and corresponded to cells with a regulatory and naïve phenotype expressing intracellular CTLA-4 and CD122. The remaining one-third of the CD25^+^ T cells were CD45RA^−^, CD62L^low^, CD38^low^, CD122^low^, CTLA-4^−^ and might constitute effector T cells [18]. However, CB CD25^+^CD45RA^−^ T cells expressed *foxp3* mRNA although at lower levels than T cells with a regulatory phenotype [19]. As these data were obtained by quantitative RT-PCR assay for *foxp3* mRNA, it was possible that the level of *foxp3* mRNA did not directly correlate with the CB Treg function. Regarding this issue, we sorted the CD25^+^ T cells by FACS into subpopulations differing in intracellular FOXP3 protein levels. As shown in Figure 5(c), we observed that CB FOXP3^+^CD4^+^ T cells, which were all FOXP3^low^, consisted mainly of CD45RA^+^ T cells, and that APB FOXP3^low^ CD4^+^ T cells did not coexpress CD45RA^+^ at all. These findings would suggest a potential relationship between the change in FOXP3 expression and CD45 isoform switching during T cell development.

Regarding the relevance of our findings to fetomaternal immunology, it is an issue that needs to be considered based on our observation that the suppressive activity of CB CD25^+^CD4^+^ T cells was at a very low level compared to that of APB CD25^+^CD4^+^ T cells (Figures 2 and 3). The placental barrier does not completely separate fetus and mother, with immunocomponent cells being able to cross this barrier in both directions [34]. The immunotolerant behavior of the mother toward the fetus is known to be necessary for survival, and it is equally important for the fetus torecognize and eliminate maternal immunocomponent cells as immunologic defense against maternal cell attack [35, 36]. It is therefore a reasonable possibility that functional downregulation of CB Treg is associated with fetomaternal immune balance in pregnancy. On the other hand, the suppressive activity of CB Treg is exerted after stimulation of CB CD4^+^CD25^+^ T cells via TCR plus CD28 signaling (Figure 6). This result may explain the observation that HLA-mismatched allogeneic transplantation, which can result in severe graft-versus-host disease (GVHD) in bone marrow or peripheral blood stem cell transplantation, is possible in cord blood transplantation with tolerable GVHD [37]. Moreover, an increase in CB Treg suppressive activity following cellular stimulation may reduce the risk of developing autoimmune and allergic diseases after birth.

## Figures and Tables

**Table 1:** Combinations of antibodies used in FACS analysis.

	Fluorophore	FITC	PE	PE-TR	PerCP-Cy5.5	PE-Cy-7	APC	APC-Cy7	Pacific Blue
Surface staining^(a)^	Ag	CD45RO	CD25	PI	CD8	CD3	CD62L	CD4	CD45RA
	Clone	UCHL1	4E3		SK1	SK7	Dreg56	RPA-T4	MEM-56

Intracellular staining^(b)^	Ag	CD62L	CD152	EMA	CD8	CD4	CD25	-	CD45RA
	Clone	Dreg56	BNI3		SK1	SK3	M-A251		MEM-56
	Ag	CD45RA	FOXP3	-	-	CD152	CD25	CD4	CD8
	Clone	HI100	PCH101			BNI3.1	M-A251	RPA-T4	RPA-T8

^(a)^All Abs were added to cells except PI, and cells were stained for 20 minutes. PI were added just before FACS analysis.

^(b)^All Abs were added to cells except anti-FOXP3-Ab and/or anti-CD152-Ab, and cells were stained for 20 minutes. After cell fixation and permeabilization, anti-FOXP3-Ab and anti-CD152-Ab were added for intracellular staining.
